# The Characteristics and Risk Factors of Web-Based Sexual Behaviors Among Men Who Have Sex With Men in Eastern China: Cross-sectional Study

**DOI:** 10.2196/25360

**Published:** 2021-09-02

**Authors:** Lin Chen, Wanjun Chen, Tingting Jiang, Zhikan Ni, Qiaoqin Ma, Xiaohong Pan

**Affiliations:** 1 Zhejiang Provincial Center for Disease Control and Prevention Hangzhou China

**Keywords:** HIV, men who have sex with men, casual sexual partners, internet, cross-sectional study

## Abstract

**Background:**

Finding casual sex partners on the internet has been considered a huge challenge for HIV transmission among men who have sex with men (MSM) in China.

**Objective:**

This study aimed to identify the characteristics and risk factors of finding causal sex partners on the internet among MSM in Zhejiang Province, China.

**Methods:**

This was a cross-sectional study. Participants were enrolled by 4 community-based organizations (CBOs) and 10 Voluntary Counselling and Testing (VCT) clinics through advertisements in bathrooms, bars, and gay hook-up apps from June to December 2018. A CBO- or physician-assisted survey was conducted to collected information on finding casual sex partners, perceived HIV infection, and HIV risk behaviors.

**Results:**

Among 767 participants, 310 (40.4%) reported finding causal sex partners on the internet. Factors associated with finding casual sex partners on the internet included watching pornographic videos on the internet more than once a week (adjusted odds ratio [aOR]=1.881, 95% CI 1.201-2.948), discussing “hooking-up online” with friends (aOR=4.018, 95% CI 2.910-5.548), and perceiving that the likelihood of HIV infection among casual sex partners sought on the internet was “medium” (aOR=2.034, 95% CI 1.441-2.873) or “low” (aOR=2.548, 95% CI 1.524-4.259). Among the participants who reported finding casual sex partners on the internet, 30.2% (91/310) reported having unprotected sex with casual sex partners encountered on the internet in the past 6 months. On multivariate logistic regression analyses, knowing the HIV infection status of casual sex partners sought on the internet was significantly associated with performing inserted intercourse (aOR=1.907, 95% CI 1.100-3.306) and a decreased risk of inconsistent condom use (aOR=0.327, 95% CI 0.167-0.642).

**Conclusions:**

Web-based casual sexual behavior is becoming more prevalent, and the rate of unprotected sex among MSM in Zhejiang Province is high. Future HIV prevention approaches should emphasize the importance for MSM to proactively determine the HIV infection status of potential casual sex partners sought on the internet.

## Introduction

Globally, men who have sex with men (MSM) continue to be disproportionately affected by HIV [1,2]. Homosexual behavior has been the main route of HIV transmission in China. In 2019, HIV infection through male-male sexual contact accounted for 40% of all cases of HIV infection in Zhejiang Province (data not published). There are many risky factors of HIV infection among MSM, such as substance abuse, multiple sex partners, and sex position [3,4]. One challenge to the prevention of HIV transmission among MSM is the increasing trend of finding casual sex partners on websites and smartphone apps [5].

The internet’s role as a platform enabling MSM to engage with other men for both social and sexual purposes began with the establishment of web-based chat rooms in the late 1990s in the United States [6,7]. Popular hook-up apps accessible on smartphones include Grindr, Jack’d, Manhunt, Scruff, and Black Gay Chat in the United States [7,8]. In China, Blued is the most popular hook-up app among MSM; it was launched in 2009 and has more than 30 million registered MSM users, accounting for roughly 4.1% of all adult men in China. The number of MSM registered as users on Blued were 409,000 in Zhejiang Province, China [9].

With the rapid increase in popularity of hook-up apps, more MSM are finding casual sex partners on the internet. The benefits of finding casual sex partners on the internet include greater convenience, accessibility, and anonymity. The rate of MSM finding sex partners on the internet ranged from 30% to 86% in different countries [10,11]. Finding casual sex partners on the internet was associated with HIV infection [5]. Studies on the characteristics and the difference of risky behavior between web-based groups and offline groups are rare.

There has been no definite conclusion about the reasons for finding casual sex partners on the internet and for condom use among sex partners sought on the internet. Serosorting was an effective strategy, first acknowledged in the 1990s [12,13]. A Joint United Nations Programme on HIV/AIDS report indicated that condom use with other men without regard to HIV serostatus was the only major risky behavior among MSM [14,15]. The Chinese Center for Disease Control and Prevention released a guide for preventing HIV among MSM (2016 version), which emphasizes educating MSM on the importance of proactively determining the HIV infection status of sexual partners. However, users are not required to disclose their HIV infection status on Blued or other apps, which affects decision-making regarding condom use. Little is known about the effect of this strategy on condom use with casual sex partners sought on the internet.

To explore the status of individuals finding casual sex partners on the internet and to examine the factors related, we investigated the characteristics of MSM seeking casual sex partners on the internet and compared the risky behavior between a web-based group and an offline group. We also investigated whether peer communication, perceived risk of HIV infection, alcohol consumption, and exchange of information regarding HIV infection status were associated with the risky sexual behavior.

## Methods

### Study Population

This cross-sectional study examined MSM between June and December 2018 in Zhejiang Province. Criteria for enrollment were males who (1) have had anal or oral intercourse with a male within the past 6 months, (2) were aged 18 years and older, (3) resided locally for more than 6 months, and (4) consented to participate in the study.

### Study Design and Data Collection

Subjects were enrolled by 4 local CBOs and 10 Voluntary Counselling and Testing (VCT) clinics in Zhejiang Province through venues for gay men and networks formed by gay men. They serve more than 50% of all MSM in Zhejiang Province and are located in the cities of Hangzhou, Ningbo, Wenzhou, Shaoxing, and Taizhou. They placed advertisements regarding the study in bathrooms, bars, and in chat groups on Blued, WeChat, and Tencent to target MSM. A CBO- or physician-assisted survey was conducted through an electronic questionnaire. All participants were asked to scan a 2D code and were directed to the electronic questionnaire. All participants received face-to-face or telephone training on the questionnaire. Electronic informed consent was obtained before beginning the survey. Participants received a gift worth 30 RMB (approximately US $5.00) for completing the investigation. Cellphone numbers were used to filter duplication, and no duplicated participants were found.

We calculated the sample size on the basis of the rate of finding sexual partners on the internet, which ranges from 40% to 60%. The minimum sample size required for this study was estimated at 266 people, with a Cronbach α of .10 and β value of .10, calculated using PASS (version 11.0, NCSS, LLC).

In total, 812 individuals participated in this study. Of these, 793 (97.7%) were eligible to participate during the data collection period, 26 of whom did not complete the survey. Ultimately, 767 participants were enrolled in this study.

Two questions were asked to evaluate “finding casual sex partners online” or “offline”: “Have you ever dated and had sexual intercourse with men you met on the Internet, such as with Blued, WeChat, a chat room, or other?” and “Have you ever have sexual intercourse with men you met in a bar, park, bathing pool, or other place?”

One question was asked to evaluate “Knows HIV epidemic”: “Are MSM the most seriously affected by AIDS in China at present?” For the logistic regression analyses, replying with “No” or “unknown” was defined as “No.” Inconsistent condom use was deemed as “ever have sex intercourse with no condom.”

### Ethics Approval and Consent for Publication

All procedures performed in the study were approved by the Ethics Committee of Zhejiang Provincial Center for Disease Control and Prevention (2018-033). This study did not involve any animals. All participants signed electronic inform consent.

### Statistical Analysis

We used SPSS (version 19.0, IBM Corp) to analyze the data. Descriptive analyses were used to describe the demographic characteristics of all subjects finding casual sex partners on the internet. The chi-square test was used to examine the differences between proportions in accordance with the studied characteristics. We performed univariate and multivariate logistic regression analyses (Backward: LR) to identify the independent risk factors associated with finding casual sex partners on the internet and inconsistent condom use with casual sex partners sought on the internet. All variables were included in the model. Missing data were not included in the analysis. *P* values of <.05 were considered to indicate statistical significance.

## Results

The demographic and behavioral assessments included 767 MSM. Of them, 76.1% (585/767) were aged 16-34 years, and 62.0% (476/767) had a college education or above. A total of 422/767 (55.0%) were registered residents in Zhejiang Province, and 54.2% (416/767) had lived locally for more than 5 years. A total of 227/767 reported annual incomes exceeding 100,000 RMB (US $14,700). Among all subjects, 62.2% (477/767) self-identified as gay and 31.7% (243/767) as bisexual. Among the 767 MSM, 310 (40.4%) had met at least 1 partner on the internet in their lifetime. (Table 1).

**Table 1:** Sociodemographic characteristics of all men who have sex with men and those who found partners on the internet (N=767).

Characteristics		All men who have sex with men (n=767), n (%)	Men who have sex with men who found partners on the internet (n=310), n (%)
**Age (years)**			
	16-24	248 (32.3)	105 (33.9)
	25-34	337 (43.9)	141 (45.5)
	≥35	182 (23.7)	64 (20.6)
**Education level**			
	High school or below	291 (38.0)	115 (37.1)
	College or bachelor’s degree	449 (58.5)	176 (56.8)
	Master’s degree or doctorate	27 (3.5)	19 (6.1)
**Registered permanent residence**			
	Zhejiang Province	422 (55.0)	183 (59.0)
	Other provinces	345 (45.0)	127 (41.0)
**Length of local residence** **(** **years)**			
	0-1	47 (6.1)	30 (9.7)
	1-3	190 (24.8)	75 (24.2)
	3-5	114 (14.9)	37 (11.9)
	≥5	416 (54.2)	168 (54.2)
**Annual income (10,000 RMB)^a^**			
	0-5	247 (32.2)	96 (31.0)
	5-10	293 (38.2)	120 (38.7)
	≥10	227 (29.6)	94 (30.3)
**Sexual orientation**			
	Gay	477 (62.2)	205 (66.1)
	Bisexual	243 (31.7)	95 (30.6)
	Heterosexual/unsure	47 (6.1)	10 (3.2)

^a^1 RMB=US $0.15.

Of the 310 MSM who found casual sex partners on the internet, 62.9% (195) found partners only on the internet and 37.1% (115) found partners both on the internet and offline. Overall, 93.5% (290/310) of these MSM found partners using Blued, as opposed to 19.4% (60/310) of those who used other hook-up apps, and 8.4% (26/310) using social apps or websites. More than one-third (60.4%, 177/293) had sexual intercourse with casual sex partners sought on the internet at a hotel, karaoke lounge, or club, and 90.0% (269/299) of them dated in their local city. Among MSM who found partners on the internet, 24.6% (91/301) reported inconsistent condom use in the past 6 months with casual sex partners sought on the internet (Table 2).

Compared to MSM who found partners only on the internet, those who found partners both on the internet and offline were more likely to report ≥2 web-based dates per month (76.3%, 87/114 vs 54.6%, 106/194; *P*<.001), ≥2 casual sex partners sought on the internet (79.2% 84/106 vs 68.5%, 126/184; *P*=.048), inconsistent condom use with casual sex partners sought on the internet (39.5% 45/114, vs 24.6%, 46/187; *P*=.006), and no condom use during intercourse after drinking alcohol (19.1% 22/115, vs 5.2%, 10/184; *P*=.001) (Table 2).

**Table 2:** Association between seeking casual sex partners on the internet in the past 6 months and the frequency of dating, condom use, and location where sexual intercourse occurred among men who have sex with men who found partners on the internet only or both on the internet and offline (N=310).

Variables		Men who have sex with men who found partners only on the internet, n (%)	Men who have sex with men who found partners on the internet and offline, n (%)	Total	Chi-square (*df*)	*P* value
**Frequency of finding partners on the internet (times/month) in the past 6 months**					14.421 (*1*)	<.001
	1	88 (45.4)	27 (23.7)	115		
	≥2	106 (54.6)	87 (76.3)	193		
	Missing	1	1	2		
**Number of casual sex partners sought on the internet in the past 6 months**					3.903 (*1*)	.048
	1	58 (31.5)	22 (20.8)	80		
	≥2	126 (68.5)	84 (79.2)	210		
	Missing	11	9	20		
**Condom use with casual sex partners sought on the internet in the past 6 months**					7.429 (*1*)	.006
	Every time	141 (75.4)	69 (60.5)	210		
	Sometimes/never	46 (24.6)	45 (39.5)	91		
	Missing	8	1	9		
**Place where sexual intercourse occurred with casual sex partners sought on the internet in the past 6 months**					4.448 (*1*)	.04
	Hotel, karaoke lounge, or club	102 (55.7)	75 (68.2)	177		
	Home	81 (44.3)	35 (31.8)	116		
	Missing	12	5	17		
**City where sexual intercourse occurred with casual sex partners sought on the internet in the past 6 months**					3.426 (*1*)	.06
	Local city	172 (92.6)	97 (85.8)	269		
	Other cities	14 (7.4)	16 (14.2)	30		
	Missing	9	2	11		
**Sexual intercourse with men who have sex with men without a condom after drinking alcohol in the past 6 months**					15.191 (*1*)	.001
	No	184 (94.8)	93 (80.9)	277		
	Yes	10 (5.2)	22 (19.1)	32		
	Missing	1	0	1		
**Sexual intercourse with men who have sex with men without a condom after watching erotic videos in the past 6 months**					0.746 (*1*)	.39
	No	171 (88.6)	98 (85.2)	262		
	Yes	22 (11.4)	17 (14.8)	39		
	Missing	9	0	9		
**Number of HIV tests until now**					0.397 (*1*)	.53
	0	71 (36.4)	46 (40.0)	117		
	≥1	124 (63.6)	69 (60.0)	193		

Multivariate modeling revealed that the likelihood of finding casual sexual partners in the past 6 months was higher among MSM who watched pornographic videos on the internet more than once per week than among those who never did so (adjusted odds ratio [aOR]=1.881, 95% CI 1.201-2.948). In addition, those who discussed “hooking-up online” with friends were more likely to find partners on the internet than among those who never did so (aOR=4.018, 95% CI 2.910-5.548). Compared to MSM who perceived that the HIV infection risk from casual sex partners sought on the internet was “high,” those who perceived that the risk of HIV infection was “medium and low” were more likely to finding sex partners on the internet, with an aOR of 2.034 (95% CI 1.441-2.873) and 2.528 (95% CI 1.530-4.176), respectively (Table 3). All of the above results pertained to the past 6 months (Table 3).

**Table 3:** Uni- and multivariate logistic regression analyses of the risk factors associated with finding partners on the internet among men who have sex with men in China (N=767).

Characteristics		Met partners on the internet, % (n/n)	Odds ratio (95% CI)	*P* value	Adjusted odds ratio (95% CI)	*P* value
**Age (years)**						
	16-24	43.3 (105/248)	1	—^a^	—	—
	25-34	41.8 (141/337)	0.980 (0.703-1.366)	.90	—	—
	≥35	35.2 (64/182)	0.739 (0.498-1.096)	.13	—	—
**Education level**						
	High school or below	39.5 (115/291)	1	—	—	—
	College or above	41.0 (195/476)	1.062 (0.789-1.430)	.69	—	—
**Registry area**						
	Native	43.4 (183/422)	1	—	—	—
	Other	36.8 (127/345)	0.761 (0.568-1.018)	.07	—	—
**Sex role**						
	Receives	38.5 (77/200)	1	—	—	—
	Inserts/both	41.1 (233/567)	1.780 (0.645-1.249)	.06	—	—
**Knowledge of HIV infection**						
	Correct	41.8 (264/631)	1	—	—	—
	Incorrect/no knowledge	33.8 (46/136)	0.711 (0.482-1.048)	.09	—	—
**Watched a pornographic video on the internet in the past 6 months**						
	Never	25.0 (40/160)	1	—	1	—
	<1/week	42.0 (111/264)	2.176 (1.411-3.357)	<.001	1.565 (0.979-2.503)	.06
	≥1/week	46.4 (159/343)	2.592 (1.710-3.930)	<.001	1.881 (1.201-2.948)	.006
**Discussed the topic of finding partners on the internet with friends in the past 6 months**						
	No	24.2 (103/425)	1	—	1	—
	Yes	60.5 (207/342)	4.794 (3.515-6.537)	<.001	4.018 (2.910-5.548)	<.001
**Perceived risk of HIV infection from casual sex partners sought on the internet^b^**						
	High	28.8 (102/354)	1	—	1	—
	Medium	49.3 (149/302)	2.406 (1.743-3.321)	<.001	2.034 (1.441-2.873)	<.001
	Low	53.3 (49/92)	2.815 (1.760-4.503)	<.001	2.528 (1.530-4.176)	<.001

^a^—: not determined.

^b^Missing data: perceived risk of HIV infection among casual sex partners sought on the internet=19.

Our study also evaluated factors correlated with condom use with casual sex partners sought on the internet. On univariate and multivariate logistic regression analyses, factors independently associated with inconsistent condom use with casual sex partners sought on the internet in the past 6 months included performing inserted intercourse (aOR=1.907, 95% CI 1.100-3.306) compared with performing receptive intercourse only and knowing the HIV status of most or all of casual sex partners sought on the internet (aOR=0.327, 95% CI 0.167-0.642) compared to those who do not know or know the status of only some of their casual sex partners sought on the internet (Table 4).

**Table 4:** Uni- and multivariate logistic regression analyses of the risk factors associated with inconsistent condom use with casual sex partners sought on the internet among men who have sex with men in China (N=301).

Characteristics		Inconsistent condom uses with casual sex partners sought on the internet, % (n/n)	Odds ratio (95% CI)	*P* value	Adjusted odds ratio (95% CI)	*P* value
**Age (years)**						
	16-24	23.8 (24/101)	1	—^a^	—	—
	25-34	31.2 (43/138)	1.452 (0.811-2.601)	.21	—	—
	≥35	38.7 (24/62)	2.026 (1.020-4.025)	.04	—	—
**Education level**						
	High school and under	37.8 (42/111)	1	—	—	—
	College and above	25.8 (49/190)	0.571 (0.345-0.944)	.03	—	—
**Registry area**						
	Native	26.1 (46/176)	1	—	—	—
	Other	36.0 (45/125)	1.590 (0.968-2.612)	.06	—	—
**Knowledge of HIV infection**						
	Correct	31.8 (82/258)	1	—	—	—
	Incorrect/no knowledge	20.9 (9/43)	0.568 (0.260-1.239)	.16	—	—
**Number of casual sex partners sought on the internet in the past 6 months^b^**						
	≤2	26.6 (46/173)	1	.09	—	—
	˃2	36.0 (40/111)	1.555 (0.931-2.600)	—	—	—
**Sex role**						
	Only receives	25.5 (51/200)	1	.01	1	.02
	Inserts	39.6 (40/101)	1.916 (1.150-3.190)	—	1.907 (1.100-3.306)	—
**Watched pornographic videos on the internet in the past 6 months**						
	No	37.5 (15/40)	1	—	—	—
	Yes	29.1 (76/261)	0.876 (0.342-1.370)	.84	—	—
**Discussed the topic of finding partners on the internet with friends in the past 6 months**						
	No	37.8 (37/98)	1	—	—	—
	Yes	26.6 (54/203)	1.015 (0.358-0.999)	.98	—	—
**Perceived risk of HIV infection from casual sex partners sought on the internet^c^**						
	High	28.3 (28/99)	1	—	—	—
	Medium/low	32.1 (62/193)	0.329 (0.705-2.042)	.18	—	—
**Knows HIV status of casual sex partners sought on the internet in the past 6 months^d^**						
	None/some	35.4 (75/212)	1	.001	1	.001
	Most/all	16.3 (14/86)	0.355 (0.188-0.672)	—	0.327 (0.167-0.642)	—

^a^—: not determined.

^b^Missing number of casual sex partners sought on the internet=17.

^c^Missing data: perceived risk of HIV infection from casual sex partners sought on the internet=9.

^d^Missing number of individuals who know the status of casual sex partners sought on the internet in the past 6 months=3.

## Discussion

### Principal Findings

This study found that 39% of MSM reported finding partners on the internet, and 30.2% reported having unprotected sex with sex partners sought on the internet. The factors related to sexual behavior over the internet and unprotected sexual behavior were also explored in this study.

Finding casual sex partners on the internet became popular among MSM in recent years in China. The proportion of finding partners on the internet was lower this study than in many other studies in China and other countries [16-18]. Hook-up apps for MSM have only recently become popular in China, so the proportion of individuals engaging in web-based dating is not as high as in Europe and the United States. We identified some notable characteristics of web-based hook-ups: for example, two-thirds of MSM found partners on the internet only and one-third found partners both on the internet and offline; most MSM met partners they found on the internet at a hotel, karaoke lounge, or club. This information suggests that the intervention can be complemented through apps and hotel visits.

Previous studies have revealed that MSM who find partners on the internet were more likely to engage in risky sexual behaviors [19]. This study revealed an important outcome that the frequency of casual sexual behavior, number of casual sex partners, and sex without a condom were much higher among MSM who found partners both on the internet and offline than among those who found partners only on the internet. This result indicates the key group of individuals among those who engage in web-based dating, who need more intervention.

Discussing finding partners on the internet with friends was an important risk factor for finding casual sex partners on the internet. Based on the theory of diffusion of innovations, the behavior of an individual is influenced by other members of the same group, which is called the peer effect [20]. In this study, MSM who discussed web-based hook-ups with friends might have been influenced by their friends to behave similarly. To reduce HIV-related risky behavior, peers and CBOs should focus on sharing health-related information [21,22].

Men’s perception of the danger of their sex partners is another important variable. With the popularity of hook-up platforms and apps, people find casual sex partners on the internet because of novelty, without considering the risk to their health. Commercial sex workers are always difficult to identify if they find customers on the internet [15,23]. Furthermore, pornographic videos and electronic books have become more accessible, arousing people sexually and leading them to seek a sexual release [24]. The characteristics of pornographic videos that trigger hook-up behaviors need to be explored for further intervention.

This study also found that knowing the HIV status of casual sex partners encountered on the internet was significantly associated with safer sexual behaviors. Compared with the serosorting strategy, cognizance of the HIV infection status of partners may help people make decisions leading to safer sex [25,26]. People who share their HIV status with partners are always aware of their health. The likelihood of condom use is increased if they do not know the HIV infection status of partners found on the internet. In fact, the proportion of MSM knowing the HIV status of partners found on the internet was very low in this study and in other studies [27]. There are many reasons for this: for example, MSM usually do not carry documents showing their HIV status, where this may be perceived as a violation of their privacy. Future efforts need to focus on providing documentation regarding HIV status in MSM and encouraging them to share their HIV test results on apps before hooking up.

In this study, MSM performing insertive sex were more likely to report unprotected sexual behavior. Sexual pleasure, self-efficacy in the area of sexual control, and psychosocial health mediate differences among sexual roles in terms of condom use [28-30]. Furthermore, the proportions of MSM who use drugs for sexual pleasure increased from 5% in 2003 to 40% in 2014 [5]. Further research needs to examine the mechanism of how sexual roles impact condom use.

Although this study initially revealed the relationship between the use of hook-up apps and risky sexual behavior, it is not directly related. Sociological and psychological factors may be potential directly related as well. These associations should be explored by future studies.

### Limitations

Our study has several limitations. First, our study population might not be representative of the general MSM population in Zhejiang Province. Self-selected men who volunteered to participate in the study were recruited, so the sample was subject to selection bias. The participants completed the questionnaire in confidence, so it might have been subject to social desirability and information biases. To minimize bias, the introductory section of the questionnaire emphasized the need for commitment form the participant to ensure high-quality data. Furthermore, all questionnaires were checked once a week and revised if an input error or missing data were identified. This has been shown to reduce information bias by self-reporting. Finally, this study was cross-sectional; hence, our findings do not extend to all MSM in Zhejiang Province, and a cohort study is needed to validate these findings.

### Conclusion

Internet-based casual sexual behavior is becoming popular among MSM in Zhejiang Province. Those who found casual sex partners both on the internet and offline reported a higher rate of unprotected sexual behavior and more casual sex partners. Watching pornographic videos on the internet more than once per week, discussing “hooking-up online” with friends, and perceiving the risk of HIV infection among casual sex partners sought on the internet as “low” or “medium” were associated with finding casual sex partners on the internet. Performing insertive sex, knowing the HIV status of casual sex partners found on the internet decreased the risk of inconsistent condom use with these casual sex partners. Intervention programs are required to encourage MSM to exchange information regarding their HIV infection status with prospective sex partners. Peer education could play an important role in helping MSM consider their health and making correct decisions.

## Abbreviations

aOR: adjusted odds ratio
CBOs: community-based organizations
MSM: Male who have sex with male
VCT: Voluntary Counselling and Testing
