# Correction: The Characteristics and Risk Factors of Web-Based Sexual Behaviors Among Men Who Have Sex With Men in Eastern China: Cross-sectional Study

**DOI:** 10.2196/33430

**Published:** 2021-09-08

**Authors:** Lin Chen, Wanjun Chen, Tingting Jiang, Zhikan Ni, Qiaoqin Ma, Xiaohong Pan

**Affiliations:** 1 Zhejiang Provincial Center for Disease Control and Prevention Hangzhou China

In “The Characteristics and Risk Factors of Web-Based Sexual Behaviors Among Men Who Have Sex With Men in Eastern China: Cross-sectional Study” (JMIR Public Health Surveill 2021;7(9):e25360), one error was noted.

Due to a system error, the name of one author, Lin Chen, was replaced with the name of another author on the paper, Xiaohong Pan. In the originally published paper, the order of authors was listed as follows:

Xiaohong Pan, Wanjun Chen, Tingting Jiang, Zhikan Ni, Qiaoqin Ma, Xiaohong Pan

This has been corrected to:

Lin Chen, Wanjun Chen, Tingting Jiang, Zhikan Ni, Qiaoqin Ma, Xiaohong Pan

In the originally published paper, the ORCID of author Lin Chen was incorrectly published as follows:

0000-0003-3373-3393

This has been corrected to:

0000-0003-2197-2733

The correction will appear in the online version of the paper on the JMIR Publications website on September 8, 2021, together with the publication of this correction notice. Because this was made after submission to PubMed, PubMed Central, and other full-text repositories, the corrected article has also been resubmitted to those repositories.

